# Cognitive bias and attitude distortion of a priority decision

**DOI:** 10.1007/s10339-022-01097-y

**Published:** 2022-06-08

**Authors:** Ola Svenson, Torun Lindholm Öjmyr, Sophia Appelbom, Freja Isohanni

**Affiliations:** 1 Department of Psychology, Stockholm University, Stockholm, Sweden; 2 Decision Research, Eugene, Oregon USA; 3 Department of Learning, Informatics, Management and Ethics, Karolinska Institutet, Stockholm, Sweden

**Keywords:** Motivated reasoning, Cognitive bias, Time saving bias, Planning policy, Medical efficiency

## Abstract

The resource saving bias is a cognitive bias describing how resource savings from improvements of high-productivity units are overestimated compared to improvements of less productive units. Motivational reasoning describes how attitudes, here towards private/public health care, distort decisions based on numerical facts. Participants made a choice between two productivity increase options with the goal of saving doctor resources. The options described productivity increases in low-/high-productivity private/public emergency rooms. Jointly, the biases produced 78% incorrect decisions. The cognitive bias was stronger than the motivational bias. Verbal justifications of the decisions revealed elaborations of the problem beyond the information provided, biased integration of quantitative information, change of goal of decision, and motivational attitude biases. Most (83%) of the incorrect decisions were based on (incorrect) mathematical justifications illustrating the resource saving bias. Participants who had better scores on a cognitive test made poorer decisions. Women who gave qualitative justifications to a greater extent than men made more correct decision. After a first decision, participants were informed about the correct decision with a mathematical explanation. Only 6.3% of the participants corrected their decisions after information illustrating facts resistance. This could be explained by psychological sunk cost and coherence theories. Those who made the wrong choice remembered the facts of the problem better than those who made a correct choice.

## Introduction

Attention to relevant facts and correct use of them are crucial for well informed decision making. But, in many situations politicians and policy makers as well as individual decision makers rely on quick, intuitive and apparently reasonable judgments of facts to motivate a choice. Such simplifying judgments and decision rules or heuristics are often used when the correct way of using available information is unknown, too complex or when time is too short for correct processing. Heuristics facilitate but can also systematically bias decision processes and many researchers have investigated different cognitive heuristics (Gigerenzer 2008; Gigerenzer and Todd 1999a,b; Gilovich et al. 2002; Kahneman et al. 1982; Kahneman and Tversky 1982; Svenson 1970, 2008). In the present study, we will investigate the effects of two heuristics with and their biasing effects on a decision. The first is the motivated reasoning bias which describes how motivation and attitudes distort a decision that should be based on facts and explicit values only (Kunda 1990). The second bias, the resource saving bias, is a cognitive bias describing how resource savings from improvements of highly productive units are overestimated in comparison with improvements of less productive units (Svenson 2011). The present study explores if and to what extent the resource saving bias in combination with motivated reasoning distorts a decision in the health sector. The participants will be asked to take the role of a policy maker and will be asked about reasons for their decisions. In the following, we will first introduce motivated reasoning and then the resource saving bias.

### Attitude and motivated reasoning

Kunda (1990) draws attention to two different goals in decision making, accuracy and motivational goals. Motivational goals describe wishes, desires or preferences that concern the outcome of given reasoning task. Kunda explains that people who are motivated towards a particular conclusion will try to rationalize it, and form a justification in order to influence others to come to the same conclusion. They draw the intended conclusion only if they can present persuasive evidence to support it (Kunda 1990). However, motivated goals may dominate and distort decisions even if factual information is available suggesting otherwise (Allison and Messick 1985). Hahn and Harris (2014) give an extensive and detailed review of biases and motivated reasoning from 1906 until 2013. Motivated goals include, for example, evaluative attitudes towards a group, actors, activities, objects and products and these goals have been studied by researchers specializing in social and cognitive psychology, economy and political science etc. (Baekgaard et al. 2020; Donovan and Priester 2017; Epley and Gilovich 2016; James and Van Ryzin 2017; Kahan et al. 2017; Redlawsk 2002). Redlawsk (2002) investigated political decision making in an experimental setting. He found that participants who indicated that they used motivated reasoning increased their support for a positively evaluated candidate after learning about new negatively evaluated information about their own candidate. Baekgaard and colleagues (Baekgaard 2019) investigated 954 Danish politicians who made fictitious decisions based on facts (number of satisfied parents) about private and public schools. They found politically motivated reasoning and that additional facts against the incorrect motivated reasoning alternative tended to increase the support for that alternative.

With these findings in mind, we decided to test the hypothesis that additional facts against a previous choice of an incorrect motivated reasoning alternative will not lead to an increased frequency of correct choices. In the present context, we will superimpose a motivated goal on the cognitive resource saving bias. The latter leads to objectively incorrect decisions, and we will study the joint effects of the two biases on choices. A corresponding dual approach was also chosen by, for example, Kahan and colleagues (Kahan et al. 2017) and Lind and colleagues (Lind et al. 2018) when they investigated the cognitive base rate bias jointly with a motivated reasoning goal. To exemplify, Lind et al. presented a 2 × 2 table with frequencies of number of Norwegian communities that received/did not receive refugees crossed with increase/decrease of crime rate. The numbers in the tables induced the cognitive base rate neglect bias, which was reinforced by a motivated reasoning bias based on attitude. The results showed the combined effects of these biases and indicated that higher numerical ability seemed to reduce some of the effects of motivated reasoning.

### The cognitive resource saving bias

The resource saving bias (Svenson 2011) is a bias that was derived from the time saving bias (Svenson 1970, 2008), and the underlying mathematical relationships are the same. The latter bias describes how the time saved following speed increases from already high speeds are overestimated compared to time saved after increases from low speeds. In the resource saving, bias speed corresponds to productivity and time to man-hours or another production resource. Svenson (2011) asked participants to choose one of a company's two manufacturing sites for an investment to increase productivity. The purpose of the investment was to save production resources for other activities. Productivity is measured in number of units produced per hour, units produced by one machine or worker per day, etc., and the participants were given information including the following: *An industry is about to decide in which of two production lines to invest for greater productivity, the line at site A or at B. The two lines produce the same product and the same number of the product (10 000 each) but at different sites. Your task is to choose the alternative A or B, which after a productivity improvement (same cost for both sites) will save most production time resources (hours) compared to the situation before the production improvement.*

**Table:** 

	A	B
Original productivity	30 units/h	70 units/h
Improved productivity	40 units/h	110 units/h

As predicted, Svenson (2011) choices of resource savings following a production speed increase from a low production speed line were underestimated in comparison with an increase from a high production speed. This means that most people prioritized the productivity improvement of B which is incorrect and illustrates the resource saving bias. It is interesting to note that this bias belongs to a group of biases related to the time saving bias (Svenson 1971) and include time saved by driving faster (Peer 2010; Peer and Gamliel 2012; Svenson 2008), time lost by decreasing speed (Svenson and Treurniet 2017; Svenson and Borg 2020), health care efficiency (Svenson 2008), well-fare costs (Tscharaktschiew 2016), consumer products (De Langhe and Puntoni 2016) and fuel efficiency of cars (Larrick and Soll 2008). People do have an idea about the problems in these contexts and are ready to make intuitive judgments; however, they are biased in most cases.

In the present study, we will compare subjective judgments with objective facts. Therefore, we will present the objective formula for computing resource savings (e.g. man-hours), which includes differences between measures of productivity. Such differences are intuitively very problematic to judge for most people (Eriksson and Jansson 2016; Svenson 2021; Svenson and Borg 2020). The difference in Eq. (1) gives the normatively correct decision parameter D for a choice between sites A and B. If D > 0.0, alternative A saves most resources. R_A2_ and R_A1_ represent the improved and original productivity (production speeds) for the A site and R_B2_ and R_B1_ the corresponding variables in units per hour for the B site. P is the total production at each site, 10 000 units.

1 $${\text{D }} = {\text{ P}}\left[ {\left( {{1}/{\text{R}}_{{{\text{A1}}}} - { 1}/{\text{R}}_{{{\text{A2}}}} } \right) \, - \, \left( {{1}/{\text{R}}_{{{\text{B1}}}} - { 1}/{\text{R}}_{{{\text{B2}}}} } \right)} \right]$$

In this example, calculation of the resources of production time saved for A gives 10 000x(1/30−1/40) = 83 h. And the corresponding calculation for B gives 10 000x(1/70−1/110) = 52 h, which is only 63% of the 83 h saved by the low level productivity improvement.

What do people do when they make the resource saving bias? According to Eq. (1), the resource saved for alternative A can be written in the following form.

2 $${\text{Resource saved A }} = {\text{ P}}\left[ {\left( {{\text{R}}_{{{\text{A2}}}} - {\text{ R}}_{{{\text{A1}}}} } \right) \, / \, \left( {{\text{R}}_{{{\text{A1}} }} {\text{x}} {\text{R}}_{{{\text{A2}}}} } \right)} \right]$$

Svenson and colleagues (Svenson et al. 2014) showed that participants did not use the denominator (R_A1_ x R_A2_) properly and that they made intuitive judgments as if one or both of the factors in the denominator were constant. When one of these factors is constant (e.g. 1.0), judgments follow a proportional change rule and when both are constant the judgments follow the difference between production speeds.

Why do people make systematic errors like the resource saving bias? It is obvious that the function in Eq. (1) is difficult to judge and therefore simpler heuristics are used. Assuming that a numerical judgment precedes a decision, the Numerical Judgment Process Model, NJP describes the cognitive processes activated when a problem is interpreted and processed (Svenson 2016).

In particular, the theory predicts that additive and linear functions will be attempted first before curvilinear or multiplicative functions (Svenson 2016). The model states that one reason for this is the great power of additive and linear functions to also approximate curvilinear functions in the natural environment and that people learn this which makes additive, difference and linear functions readily available when a problem appears. Also, NJP postulates that the cognitive effort needed for performing additive strategies is generally smaller than the cognitive effort needed for, for example, multiplicative strategies like estimations of proportions or percentages. This is because proportion judgments include both a difference and a ratio calculation. Stanovich (2018) would describe the activation of the different strategies as elicitation of mindware in automated System 1 associations.

### Biased decisions, implementation, outcomes and motivated reasoning

Most studies of cognitive biases are silent about who would make the decision, implement and pay for the implementation and information about who will be affected by the consequences of the decision (Gilovich et al. 2002; Gigerenzer 2008; Gigerenzer and Todd 1999a,b; Kahneman et al. 1982; Kahneman and Tversky 1982; Svenson 2008, 2011, 2016). To exemplify, there are seldom different groups of people who die from the Asian disease in the framing problem introduced by Tversky and Kahneman (1981) and no costs included (Kühberger 1998). When a participant in a study does not know who would make and take responsibility for a decision, implement it, pay for the implementation and who will experience the outcomes of the decision, the problem is underspecified for well informed decision making. In a recent paper, Fischhoff and Broomell (2020) remarked that decision studies often do not give the information that a decision maker needs to know for a decision. With this in mind, we wanted to take a step towards a richer specification of a decision problem by identifying the different actors who will implement the decision and who will pay for the implementation. Our participants will take a role like that of a politician or a policy maker.

Knowledge about the different actors who implement a decision can activate motivated reasoning which can distort the decision. In Sweden, privatization of medical care has been massive, while the costs for treatment are still covered by society. This has split the public in terms of attitude towards public versus private for-profit medical care, and we will study how this attitude difference towards the actors may motivate the participants' decisions (Rheu 2020). We predict that such attitudes towards private and public medical care will elicit motivated reasoning and distort decisions because the importance of facts are downplayed. To specify, we will present two alternative emergency room clinics in our experiment. One of them is publicly run, and the other run by a private for-profit hospital. We will measure the participants' attitudes towards these two types of medical care providers. We will also apply the Cultural Cognitive Worldview Scales, CCWS (Kahan et al. 2011) which aims at describing individualism versus egalitarianism. We included this scale because individualism is correlated with attitudes towards private and public health care as shown by, for example, Baekgaard and colleagues (Baekgaard, et al. 2020). Cognitive reflection test, CRT, items were also included in the study because mathematical/logical ability has been shown to correlate negatively with motivational bias (Lind et al. 2018).

Based on the empirical and theoretical findings presented in Introduction, we predict (1) that a majority of participants will make the resource saving bias, (2) that the motivated reasoning bias will affect choices, (3) that the motivated reasoning bias will add to the resource saving bias when the motivated and cognitive biases both favour the same alternative, (4) that some decision makers may not change an incorrect decision after additional facts explaining that it was wrong (Redlawsk 2002; George et al. 2017). (5) We will also investigate if participants who made the correct or the wrong decision from the beginning will remember facts more or less accurately. (6) For those who made a first incorrect decision, we will investigate if the verbal justifications for a second decision are different from those before information about the correct choice. Hence, we have empirically grounded facts for predictions (1) to (4) and are open for different outcomes of the exploratory investigations (5) and (6).

## Experiment

### Method

#### Participants

In all, 365 students were approached in common spaces at the Royal Institute of Technology and Stockholm University and invited to participate in the study. Of these, 125 participants chose not to complete the questionnaire available on the Internet. In addition, 36 participants who did not answer an attention test item correctly were excluded. The final sample was 204 participants of which 106 (52%) were women, 95 (46.6%) men, and 3 (1.5%) declined to indicate a binary gender. The age of the participants ranged between 18 and 56 years with a mean of 24.56 years (SD = 6.26), and the median educational level was high mostly including some university courses. The participants were given a candy bar when approached and a link to the questionnaire. After having filled out the questionnaire, each participant received an electronic lottery ticket.

#### Material and procedure

The questionnaire started with problems from the Cultural Cognitive Worldview Scales individualism and egalitarianism (Kahan et al. 2011) with Cronbach's α = 0.70 for the individualism and α = 0.86 for the egalitarianism scale. This was followed by 7 items about attitudes towards publicly driven and private for-profit driven health care. Attitude towards medical care actors (private for-profit or public) running Swedish tax financed health care services was measured for seven items on 7-point Likert scales (1 = Do not agree at all; 7 = Completely agree) with Cronbach’s α = 0.80 in Study 1. The items were developed by the authors and aimed to measure attitudes towards government funded for-profit run versus publicly non-profit run health care. The scale contained the following items (in Swedish). (1) The best for Sweden is that health care financed by the tax payers is run by Landstingen (the local state district organization that collects taxes for health care), (2) the best for Sweden is that health care financed by the tax payers is run by private for profit companies, (3) health care financed by the tax payers and run by private for-profit companies should be supported, (4) it should not be possible for private for-profit companies to make unlimited profit from health care financed by the tax payers, (5) tax financed health care run by Landstingen is often poorer than the same health care run by private for-profit companies, (6) tax financed health care run by private for profit companies should be prohibited, and (7) health care financed by the tax payers and run by Landstingen should be supported.

After this, the main decision problem followed with the following instruction (translation from Swedish).

"The health authorities have found that the long waiting times in emergency rooms remain……

One way of supporting doctors so that they become more efficient is to reorganize so that the doctors can focus on their focal medical tasks and free them from administrative and other non-medical activities. Increased doctors' efficiency will free some doctors who can be used to shorten the long waiting times in an emergency room.

Two very big emergency rooms at hospital P and L each treat 5000 patients per month, but their efficiency differs. Both are paid by the local government, by tax money. One of the emergency rooms P, is private and owned by an international private equity company for profit and the other L, by the local government with no profit. Both emergency rooms have plans for efficiency improvements that will be paid by the local government (buildings, support systems, etc..). The local government has resources to implement only one improvement plan. We know the numbers of patients treated by a doctor on average during 10 h before and after an improvement.

Below, you find a table describing the situation in each of the emergency rooms (L for public) and P (for private), the present situation and the situation after an improvement of each of the emergency rooms.

We ask you to select the emergency room, that you think should be given the improvement resources for a reduction of the administrative burden of the doctors, so that more time could be spent with the patients and reduce the waiting times in the emergency room".

" The following alternative should be chosen to maximally increase the overall efficiency"

Table 1 gives an example of the priority problem presented to the participants. The numbers were always the same, but the order of the labels local government and private was balanced. In this problem, the resource saving bias favours P, while the correct decision is that an improvement of L will save more doctor's time. Within the experimental and control groups, half of the participants received a scenario as in Table 1 and the other half with the L and P labels (but not the numbers) switched in a balanced design.

**Table 1:** The priority problem. Which alternative will save more doctor time?

	L	P
	Local government without profit	Private equity company with profit
	Patients during 10 h	Patients during 10 h
Now	15 patients/doctor	25 patients/doctor
After improvement	30 patients/doctor	55 patients/doctor

After a decision, the participants were asked to judge the given facts in terms of how much they supported a choice of P and L, respectively (*1* = *minimal, 7* = *maximal*). After that, they judged the importance for the decision of each of 4 efficiency measures (15, 30, 25 and 55) in Table 1 (*1* = *not at all important, 7* = *maximally important*). All participants were also asked to write down three justifications for the choice they would use if asked to justify the decision to a peer.

**Table 2:** Choice frequencies first decision

Scenario	L choice	P choice	Sum
LP	31 (15.20%)	**70** **(34.31%)**	101
PL	**90** **(44.12%)**	13 (6.37%)	103
Sum	121	83	204

Bold digits indicate biased choices

After having finished these judgments, the participants were asked to make a new decision. The group was divided in an experimental and a control group. The experimental group was given detailed instructions about how to calculate the number of doctor's hours that would be saved in each of the alternatives (Appendix A). The control group was given no information but some trivia questions. This section was followed by some more trivia questions that both groups received. After the second decision, the scales measuring support for each of the alternatives and the importance of facts were repeated followed by 3 cognitive reflection items, CRT. Cognitive reflection, CRT was measured using three items adapted to Swedish from the cognitive reflection test (Frederick 2005). (1) A bat and a ball cost $110 in total. The bat costs $100 more than the ball. How much does the ball cost? (2) In a lake, there is a patch of lily pads. Every day, the patch doubles in size. If it takes 48 days for the patch to cover the entire lake, how long would it take for the patch to cover half of the lake? (3) If it takes 5 machines of a subcontractor within the car industry 5 min to produce 5 components, how long would it take 100 machines to make 100 components? Alpha = 0.69. Finally, we asked all participants to reproduce, from memory, the efficiency numbers in Table 1 and this was followed by an attention check in order to eliminate participants who did not follow the instructions to read all of the text in a problem.

## Results

### Choice frequencies

The distribution of choices in the first decision can be found in Table 2. First, a majority of the participants choose the public, L alternative 121/204 (0.593) which is significantly different from chance 50%, z = 2.66, *p* < 0.01. Second, 160 of the 204 participants (0.784) made the cognitive resource saving bias, which is a significant bias, z = 8.13, *p* < 0.001. Third, the cognitive bias was marginally weaker in the group who prioritized the L alternative (90/121) = 0.744 than in the P group (70/83) = 0.843, z = −1.699, p = 0.089. There was a gender difference because women were more correct, 27.4%, than men 15.8%, X^2^ (1,201) = 3.92, p = 0.048, effect size = 0.14. According to the verbal protocols, to be reported and analysed in full later, the difference could be explained by the more frequent reference to qualitative justifications in the female group. A total of 29.8% of the female participants motivated their judgments by qualitative justifications (categories (5)−(8) in Table 5) compared to 15.4% qualitative justifications (categories (1)–(4)) in the male group.

### Judgments, scales and choice

There were no significant differences between the P and L choice groups on the Cultural Cognitive Worldview Individualism Scale, CCWS (3.31, SD = 0.83 and 3.50, SD = 0.79, respectively). The corresponding values on the egalitarianism scale were not significantly different either (2.37, SD = 1.15 and 2.40, SD = 1.01). The correlation between these scales and the scale of attitude towards private for-profit run health care in Sweden was *r* = 0.39, *n* = 204, *p* < 0.01 for the individualism scale and *r* = 0.52, *n* = 204, *p* < 0.01 for the egalitarianism scale. In the following, we will check if the Cultural World View scale developed in the USA can predict the Swedish choices above the predictions made by the attitude scale.

Each participant judged how much the information supported a choice of each alternative (minimally, 1−maximally, 7). Table 3 shows average support judgments. As would be expected, the support for a chosen alternative was stronger when the resource saving bias supported the choice (Table 3). To illustrate, the L choice group judged the support for the L alternative in the PL session, 5.84 (1.39) and in the LP session, 3.71 (1.83) which is a significant difference, t (119) = 6.76, *p* < 0.001, effect size = 1.41 (0.95−1.84) (CEM, 2020). The P choice group judged support for the P alternative in the LP session 6.00 (1.20) and in the PL session, 4.85 (0.80) which is a significant difference, t(81) = 3.31, *p* < 0.01, effect size = 1.00 (0.38−1.60).

**Table 3:** Judgments of strength of support for a choice of L and P

Decision	*L choice N* = *121*			*P choice N* = *83*		
*Session*	L judged support	P judged support	Advantage chosen	L judged support	P judged support	Advantage chosen
*LP*	3.71 (1.83) *N* = 31	4.26 (1.93) *N* = 31	– 0.55 (3.21)	2.16 (1.16) *N* = 70	6.00 (1.20) N = 70	3.84 (2.00)
*PL*	5.84 (1.39) *N* = 90	2.41 (1.24) *N* = 90	3.43 (3.28)	3.46 (0.97) *N* = 13	4.85 (0.80) *N* = 13	1.38 (1.12)
*Mean*	5.30 (1.77)	2.88 (1.65)	2.41^**a**^ (3.02)	2.37 (1.23)	5.82 (1.22)	3.46^**a**^(2.09)

a = *p* < *0.05.*

It was surprising to find that in the L choice group the judged support for the chosen alternative (-0.55) was less than the support for the non-chosen alternative. Therefore, we identified the 14 participants who showed this relationship on the individual level. Verbal protocols showed that of these 14 participants, 4 motivated their choice with mathematical arguments related to percentages, efficiency or number of patients per doctor, 1 gave an unspecified report, 4 motivated their choices with the argument that quality is more important than quantity, and the remaining 5 expressed preference for public health care. Hence, most participants in this group motivated their choices by referring to inferred information and not to the numbers given in the problem.

We wanted to extend the analyses to individual measures collected independently of the decisions. In this way, we would be able to predict choices from attitudes and the resource saving bias and determine the relative strengths of these predictors. We used L or P choice as the binary dependent measure in a logistic regression analysis with order of presentation LP, PL (scenario), attitude towards private/public health care, the cognitive reflection test items (CRT) and the world view scale (CCWS) as independent variables. To specify, we applied a logistic forward stepwise regression analysis with dependent variable choice (*L* = 0 and *P* = 1) and centred continuous variables. The analysis explained about half of the choice variance, R square = 0.49. The βs were the following: *Scenario* =  − *3.09 p* < *0.001, Attitude* = *0.52 p* < *0.01, CRT* = *0.44 p* < *0.05 and Scenario x CRT* = −*1.03, p* < *0.01.* The CCWS was not significant, and it will not be considered any further in the analyses. Hence, regression analysis did well in predicting choice. The regression analysis also demonstrated how the independently measured attitude towards the decision agent was an important predictor of choice as was CRT. Participants who made a correct decision had a significantly lower CRT score (1.46, SD = 1.27) than participants who made an incorrect decision (2.06, SD = 1.27), t(202) = 3.23, *p* < 0.01, effect size = 0.52.

### Facts information and second decision

We had participants make a new decision with the same decision problem. Half of the participants in an experimental group were informed about how to compute the correct choice and the result of such a computation (Appendix A). The information presented the computations resulting in the number of doctors saved per 100 patients after the 15 to 30 patients per doctor which was 3.34 doctors and the saving after the 25 to 55 patients per doctor improvement was 2.19 doctors. The participants in the control group were given no such information, and instead, they performed an unrelated trivia question task. The purpose of this manipulation was to describe how factual information influences or changes a participant's new decision. The choices in the new decision can be found in Table 4.

**Table 4:** Choice frequencies second decision. Incorrect responses in bold

Scenario	Control group choice	Control group choice		Experimental group choice	Experimental group choice	
	*L*	*P*	Sum	*L*	*P*	Sum
LP	13 (11.93%)	**40** **(36.70%)**	53	12 (12.63%)	**36** **(37.89%)**	48
PL	**47** **(43.12%)**	9 (8.26%)	56	**36** **(37.89%)**	11 (11.59%)	47
Sum	60	49	109	48	47	95

In the control group, 87/109 (79.8%) made the cognitive bias very close to the result from the first decision. In the experimental group, 72/95 (75.8%) showed the bias after information about facts. There were 147 (72%) participants who made the resource saving bias in both decisions, 13 (6%) who corrected their incorrect decisions, 32 (17%) who made two correct decisions and 12 (6%) who changed from a correct to an incorrect decision. Of the 147 participants who made the incorrect decision twice, 81 (55%) belonged to the control group. It was even more interesting to find that of the 13 participants who corrected their decisions 12 (92%) belonged to the experimental group.

In summary, the results indicate that (1) once the decision was made, and (2) the choice was justified to someone else, factual information about the correct decision did not change the decision for most people. This leaves us with judgments and verbal protocols if we want to know more about the cognitive processes leading to correct/incorrect decisions and information neglect.

### Judgments, scales and choice in second decision

The choice data showed that the facts information was inefficient and this was also reflected in the judgments of support of the second decision. Therefore, we analysed the two conditions jointly and found that the advantage of L over P in the L choice group, 2.43 (SD = 2.82) was smaller than the advantage of P over L in the P choice group, 3.01 (SD = 2.40), but the difference was not significant, t(202) =  − 1.53, *p* = 0.126.^1^ When the participants were asked to make a second decision, the choice data did not differ significantly between the experimental and control groups, Chi-square (1, *N* = 204) = 0.416, *p* = *0.51*9. For illustrative purposes, we computed logistic forward stepwise regression analyses for the control and experimental groups separately. When interpreting the results, it is important to keep in mind that the number of participants in each group was only half of those in the corresponding analysis of the first decision. The control group showed the following significant predictors which explained 0.47 of the variance. The βs were for Scenario = − 2.87, *p* < 0.001, Attitude = 0.53, *p* < 0.05. In contrast to the first decision, CRT did not correlate significantly with the choices in the second decision for the control group. The corresponding values for the experimental group were Scenario = −2.46, *p* < 0.001 Scenario x CRT = −0.67, *p* < 0.05. Here, attitude played no significant role and higher CRT was correlated with greater bias.

### Verbal protocols

The choices and the quantitative scales provided information about the process leading to a decision. But, we wanted to know more about the reasons for making the different choices and therefore the form included the following question, *"If you had to justify your choice to another person, which are the three most important justifications/motivations to convince that person?".*

The responses were given as free text verbal protocols. We will focus on the first justifications in the first and second decisions, respectively. Two independent coders classified the justifications in one of the following categories: (1) efficiency, (2) percentage increase (ratio), (3) increase in number (difference), (4) greater number of patients per doctor, (5) quality is more important than quantity, (6) positive attitude towards publicly run medical care, (7) positive attitude towards privately run medical care, (8) fair distribution across hospitals, (9) other and no answer.^2^ Cohen's kappa for the codes were 0.83 for the first decision and 0.77 for the second decision. When the judges had different codes, they discussed the codes and decided on a final category.

The categories refer to different interpretations of the decision problem, some of which are incorrect and relate to one of the two biases, the time resource saving and the motivational attitude biases. Categories (1)—(4) refer to attempts to solve the problem numerically as they were presented. Categories (2) and (3), ratio and difference strategies have been found in studies of resource and time saving biases (Svenson, Gonzalez, Eriksson 2018). Categories (6) and (7) refer to a motivational attitude bias related to the actor in the decision problem. Categories (5) and (8) change the problem formulation by referring to quality of care and equality of resources across hospitals. Some protocols explicitly mentioned a lack of information for a well-grounded decision (Fischhoff and Broomell 2020). These remarks indicate that the participants were quite involved in the decision problem. The results of the coding can be found in Table 5 for the first justifications given for each of the first and second decisions.

**Table 5:** Frequencies verbal justifications for the first and second decisions. Each participant gave one report for each decision. See text for explanation of the categories

Decision	Efficiency (1)	Ratio (2)	Diff (3)	No of patients (4)	Quality (5)	Positive attitude public (6)	Positive attitude private (7)	Fair distr among hospitals (8)	Other and no answer (9)
1	29	39	19	52	22	17	0	2	24
2	26	36	24	53	18	12	0	5	30
Diff 2–1	−3	−3	5	1	−4	−5	0	3	6

Cohen's kappa for the first decision = 0.826 and for the second decision = 0.774

The first 4 categories in Table 5 contain 139/204 reports (68%), same number for both the first and second decisions. This indicates that a majority of the participants tried to solve the factual problem by using the numbers in the problem. But, most of these participants failed (Table 6). In all, 22 reports in the first decision and 18 in the second decision indicate that that quality was more important than quantity (category 5 in Table 5). A total of 17 (12 in second decision) reports in category 6 indicate a positive attitude towards publicly run health care as a justification. There were no overall statistically significant differences between the justification frequencies for the first and the second decisions.^3^ Therefore, we will focus on the verbal reports from the first decision in the following.

**Table 6:** Frequencies of verbal justifications for L and P choice in both groups, first decision

	Correct choice			Incorrect choice		
Justification category	Session LP L choice	Session PL P choice	Sum	Session LP P choice	Session PL L choice	Sum
1 Efficiency	3	1	4	12	13	25
2 Ratio	1	0	1	17	21	38
3 Diff	0	0	0	12	7	19
4 No of patients	1	0	1	18	33	51
5 Quality	11	8	19	1	2	3
6 Pos att. public	9	0	9	0	8	8
7 Pos att private	0	0	0	0	0	0
8 Fair distr hospital	2	0	2	0	0	0
9 Other no answer	4	4	8	10	6	16

Table 6 shows that a majority of the incorrect decisions were justified by some kind of mathematical argument. By way of contrast, many of the correct decisions were instead justified with reference to quality (5). Not surprisingly, the positive attitude towards public health (6) justified L choices. To test the statistical significance of the association between a mathematical type of argument and an incorrect decision, the categories were grouped in three groups of categories for the first decision. The first four categories were grouped together into one group representing an attempt to solve the problem in a mathematical way, categories 5–8 constituted the second group of categories and category 9 was the third group (containing justifications that could not be coded into another category). A Chi-square test of independence showed a significant association between type of argument and correct/incorrect decision. In all, 133 of the 160 participants who had made an incorrect decision also used a mathematical justification, Chi-square (2, *N* = 204) = 90.96, *p* < 0.001, effect size = 0.67.

### Memories of facts

If a person does not read and remember the facts of a problem, the results may depend on too little attention paid to a task. Therefore, we investigated how well the participants remembered the facts of the problems. Of the 204 participants, 159 (78%) remembered the exact efficiency numbers in the problem for both the L and P alternatives. We computed the absolute difference between the reproduced and correct efficiency measure for each of the four numbers in the problem and used the mean as an index of the precision of that person's memory of the information in the problem. Hence, zero means a perfect memory and with increasing index memory becomes poorer. We grouped the participants according to the correctness of their first and second decisions, in parenthesis. The group who had both first and second choice incorrect/incorrect had an index = 0.85 (3.69), the incorrect/correct group = 2.80 (7.65), the correct/correct group = 6.85 (15.52) and the correct/incorrect group = 1.46 (5.05). A Kruskal–Wallis test for independent samples with these groups as grouping variable and mean absolute deviation as dependent measure gave significant differences between the groups Chi-square(3) = 28.34, *p* < 0.001, effect size = 0.12. It is interesting to find that the majority of participants who belonged to the group who made two correct decisions had the poorest memory of the facts in the problem and the participants who made two incorrect decisions had the best memory of the facts. Hence, an accurate memory of facts was no guarantee for an unbiased decision and the incorrect decisions cannot be explained by poor attention to the facts.

## Discussion

With reference to the predictions made in Introduction, we found that a majority of the decision makers made the resource saving bias. Second, we also found that a motivated reasoning bias distorted the choices. Third, as predicted the frequency of biased choices increased if the resource bias was coupled with a motivated reasoning bias in the same direction. Fourth, not just a few, as predicted, but a majority of the decision makers did not change their decisions after having made a first incorrect decision, justified the decision and after that had been given the possibility to change. Fifth, participants who made the incorrect decision remembered the facts of the alternatives better than those who made the correct decision. Sixth, the justifications for a repeated decision were almost identical to those given after the first decision. In summary, the cognitive resource saving bias was stronger than the motivated reasoning bias. Participants who scored better on the cognitive test items made more incorrect decisions. Also, it was interesting to find that the female participants were more correct than the male participants. This superior performance was correlated with greater emphasis on elaborations of the task by taking qualitative attributes into consideration and not with a more adequate processing of the numerical information. Finally, it is worth mentioning that if the choices are made more randomly, this will improve decision quality because this will weaken the participants' biases.

The present study includes processing of inverse variables, Eq. (1) and such functions are difficult to process (Eriksson and Jansson 2016; Svenson and Borg, 2020). One may speculate about how to improve decisions in a situation like the one investigated here (Larrick, 2004). For example, training with correct feedback is one option. This was done for the time saving bias by giving correct answers to a set of similar problems which improved performance by learning from experience (Svenson 1971). It is also possible to instruct in great detail about how to reach a judgment with examples about the underlying function (Svenson and Treurniet 2017). The motivated reasoning bias, depending on values may be harder to correct, but stressing the purpose of a decision seems necessary, and awareness of the motivated reasoning bias should be part of any debiasing procedure.

In the following, we will focus on two interesting findings: participants who made the wrong decision remembered the facts in the problem better than those who made the correct decision, and the result that a majority of participants who did not change their decisions after information that they were wrong. The verbal protocols (Table 6) inform us that a great majority of those who made the wrong decision based their choices on the numbers in the problem. By way of contrast, those who made the correct decision used other assumed and inferred facts. Therefore most likely, those who made the correct decisions spent proportionally less time on the numbers in the problem than those who made the wrong decision and based their decisions on the numbers only. If proportionally less attention was given to the numbers in the group who made the correct decision, this may explain why the memory of the facts was poorer in that group than in the group who paid attention to the numbers only.

In the present experiment, most participants did not change their incorrect decisions when given the opportunity to change. Hence, participants who made the wrong decision were resistant to correct information, in the present case backed by a mathematical computation. One may ask if this perseverance depended on less attention paid to the task, the participants not understanding the task or that they based their decisions on the efficiency numbers following the resource saving bias. First, the fact that those who made the wrong decision remembered the facts better than those who made the correct decision suggests that sufficient attention was given to the problem. Second, we have no indication that the participants did not understand the task even though we know from the verbal protocols that the goal of the task was changed by a number of participants. Third, the verbal protocols inform us that those who made the incorrect decisions actually based their choices on the numbers in the task. The puzzling fact that those with better results on the cognitive test made worse decisions may depend on a focus on the numbers in the problem and an inability to resist the resource saving bias.

Why did those who made a mistake not correct it when informed that the decision was incorrect? In his pioneering study in organizational psychology "Deep in the big muddy: A study of escalating commitment to a chosen course of action", Staw (1976) pointed out that it is costs already invested in a project that make people reluctant to change a course of action. In the present context, costs could correspond to psychological effort in a partial explanation of the results.^4^ Psychological consistency theories, such as Festinger (1957), show that people before (Svenson, 1992, 2003) and after a decision strive towards mental coherence. In doing so, they upgrade the chosen alternative and downgrade the non-chosen alternative(s) so that the chosen alternative becomes resistant against threats, such as information challenging the decision. This offers another explanation for the perseverance in the incorrect choice in the present study and elsewhere.

## Appendix

See Tables 7, 8, 9.

**Table 7:** Information to participants in experimental group after first decision

	P	L
	Private equity company with profit	Local government without profit
	*Patients during 10 h*	*Patients during 10 h*
Now	15 patients/doctor	25 patients/doctor
After improvement	30 patients/doctor	55 patients/doctor
Number of doctors now 100 patients	***6.67***	***4.00***
Number of doctors after improvement 100 patients	***3.33***	***1.81***

The text describing the matrix was the following.

"*You will now be informed about how to combine the facts to make it easier to make a decision that maximizes the total efficiency. Assume that each centre treats 100 patients during 10 h (the reasoning is the same independently of the number of patients treated and we count doctor's time in fractions of full time).*

Then, you need (100/15) = 6.67 doctors for the ***private for-profit driven centre*** to treat 100 patients. Following an improvement, you need (100/ 30) = 3.33. ***This means a saving of 3.34 doctors.***

Today, the centre run by the ***Public organization, Landstingen*** needs (100/25) = 4 doctors and after improvement 100/55) = 1.81 doctors. ***This means a saving of 2.19 doctors****".*

**Table 8:** Coding categories in Swedish with English translations

Category (English translation)		Example of argument (English translation)
1	Effektivitet (*Efficiency*)	< Den ursprungliga effektiviteten är högre för L än för P. (*The initial efficiency is higher for L than for P*) < Effektivitetsförbättring högre för L (*Higher improvement in effeciency for L*)
2	Procentuell ökning (*Percentage increase, ratio*)	< Den procentuella ökningen är större hos P (*The percentage increase is greater for P*) < Den procentuella ökningen för P är 125% medan den för L är 100% (*The percentage increase for P is 125%, while it is 100% for L*)
3	Ökning i antal (differens) (*Increase in numbers, difference*)	< Fler patienter får hjälp—30 personer fler (L) vs. 15 personer fler (P) (*More patients receive help – 30 persons more (L) vs. 15 persons more (P)*) < Kolla på differensen före och efter för de olika alternativen. P har bättre differens. (*Look at the difference before and after for the best difference. P has a better difference*)
4	Större antal patienter/läkare (*Greater number of patients per doctor*)	< Fler patienter får vård inom privatvården. (*More patients receive treatment in the private health care*) < Det maximala antalet som får hjälp blir högre på en dag. (*The maximum number receiving help during a day will be higher*)
5	Viktigare med kvalitet än kvantitet (*Quality is more important than quantity*)	< Det är bättre att varje person får längre tid på sig hos läkaren (*It is better that each person gets more time with the doctor*) < Ökad effektivitet betyder inte bättre vård/mottagande (*An increase in efficiency does not mean better care/reception*)
6	Positiv till landstingsvård (*Positive attitude towards publicly run medical care*)	< Skattemedel bör ej gå till att förbättra ett vinstdrivande företags verksamhet (*Taxpayers’ money should not be used to improve the business of a profit-driven company*) < Att (L) är ett bättre alternativ för samhället i stort (*That (L) is a better option for society at large*)
7	Positiv till privat finansierad vård (*Positive attitude towards privately run medical care*)	–
8	Rättvis fördelning mellan mottagningar (*Fair distribution across hospitals*)	< Den totala fördelningen blir jämnare (30 vs 25) (*The total distribution is more even (30 vs 25)*) < Större total effekt om både P och L fungerar effektivt än bara en av dem (*There is a greater overall effect if both P and L are efficient, than if only one of them is*)
9	Övriga och inte svarat (*Other and no answer*)	< Moral hazard < Det finns inte tillräcklig med data för att göra en bedömning (*There is not enough data to make an assessment*)

**Table 9:** Judgments and choice new decision

Decision	L choice *N* = 108			P choice * N* = 96		
Condition/ session	L support	P support	Advantage	L support	P support	Advantage
Control LP * N* = 53	4.85 (1.46) *N* = 13	3.62 (1.61) *N* = 13	1.23 (2.62) * N* = 13	2.40 (1.71) *N* = 40	5.98 (1.54) N = 40	3.58 (2.74) *N* = 40
Control PL *N* = 56	5.81 (1.28) *N* = 47	2.70 (1.69) *N* = 47	3.11 (2.41) N = 47	3.56 (1.13) *N* = 9	4.89 (1.17) *N* = 9	1.33 (1.58) *N* = 9
Experiment LP *N* = 48	3.75 (1.66) *N* = 12	4.00 (1.76) *N* = 12	– 0.25 (2.38) *N* = 12	2.64 (1.50) N = 36	5.69 (1.49) *N* = 36	3.06 (2.35) *N* = 36
Experiment PL *N* = 47	6.03 (1.23) *N* = 36	2.75 (1.81) N = 36	3.28 (2.37) *N* = 36	4.00 (2.00) *N* = 11	4.18 (1.99) *N* = 11	0.18 (2.36) *N* = 11

## Footnotes

1 Appendix C gives frequencies of judgments for the second decision.

2 Appendix B gives the Swedish text and translations in English of the categories and provides example items.

3 There were 12 participants who changed an incorrect decision to a correct decision, and they also changed their justifications (e.g., from numerical to quality justification). Grouping the participants according to their choice patterns in the successive decisions (correct–correct; incorrect–incorrect: correct–incorrect; incorrect–correct) showed that no one of these groups had a statistically significant difference in justification frequency between the first and second decisions.

4 Thaler (1980) and Arkes and Blumer (1985) demonstrated the sunk cost effect and explained it in terms of prospect theory (Kahneman and Tversky 1979) in which a person who is already on the loss side of the value function will be willing to make more risky investments than a person who has not invested anything yet. If value is exchanged for some psychological component, the prospect theory function may explain some of the present findings.
